# Microbiota characterization of *Zostera marina* seeds at early stage development

**DOI:** 10.1128/mra.01163-25

**Published:** 2026-03-20

**Authors:** Gina Chaput, Torrance C. Hanley, Jonathan A. Eisen, E. Maggie Sogin, A. Randall Hughes, Cynthia Hays

**Affiliations:** 1 Genome Center, University of California Davis 8789, Davis, California, USA; 2 Department of Biology, Sacred Heart University 3305, Fairfield, Connecticut, USA; 3 Department of Evolution and Ecology, University of California Davis 8789, Davis, California, USA; 4 Department of Medical Microbiology and Immunology, University of California Davis 8789, Davis, California, USA; 5 Department of Molecular Cell Biology, University of California Merced 33244, Merced, California, USA; 6 Marine Science Center, Northeastern University 198850, Nahant, Massachusetts, USA; 7 Department of Biology, Keene State College 4214, Keene, New Hampshire, USA; Rochester Institute of Technology, Rochester, New York, USA

**Keywords:** environmental microbiology, plant-microbe interactions, microbial ecology

## Abstract

Understanding seagrass seed microbiomes is crucial for developing microbial-mediated methods to improve germination in restoration efforts. Here, we used 16S rRNA gene and ITS2 amplicon sequencing to characterize the bacterial and fungal communities of seeds from the model seagrass, *Zostera marina*.

## ANNOUNCEMENT

Seagrass restoration is shifting from plant transplantation toward seed deployment, but establishment success remains highly variable (1, 2). Microbial inoculation, such as coating seeds with beneficial microbes (3–5) or introducing endophytes during flowering or early development (6, 7), offers a promising approach. These early-stage microbes can persist and benefit terrestrial plants into adulthood (8–10). However, applying these techniques to seagrass is challenging due to limited knowledge of aquatic seed microbiomes (11, 12). We report bacterial and fungal community characterization in seeds from the model seagrass *Zostera marina*, providing insight for restoration and future studies.

*Z. marina* seeds were collected from Curlew Beach in Nahant, MA, (42.42009°N, 70.91553°W) in late July and early August 2014 as previously described (13). DNA was extracted from 174 samples and a no-seed negative control using a Chelex method (14) and cleaned with AMPure beads. Amplicons were sequenced by the Integrated Microbiome Resource on the Illumina MiSeq platform using 2×300 bp PE v3 chemistry following established methods (15, 16). All samples were PCR amplified for the 16S rRNA V3/V4 region (primers 314F/805R), and 43 samples were PCR amplified for ITS2 (primers ITS86F/ITS4R). Sequences were analyzed in R (v4.2.1) (17) with phyloseq (v.1.42.0) (18) and DADA2 (v1.26.0) (19) using default parameters except 5’ reads were trimmed 17 bp and truncated at 290 bp, while 3’ reads were trimmed 21 bp and truncated at 200 bp.

**Fig 1:**
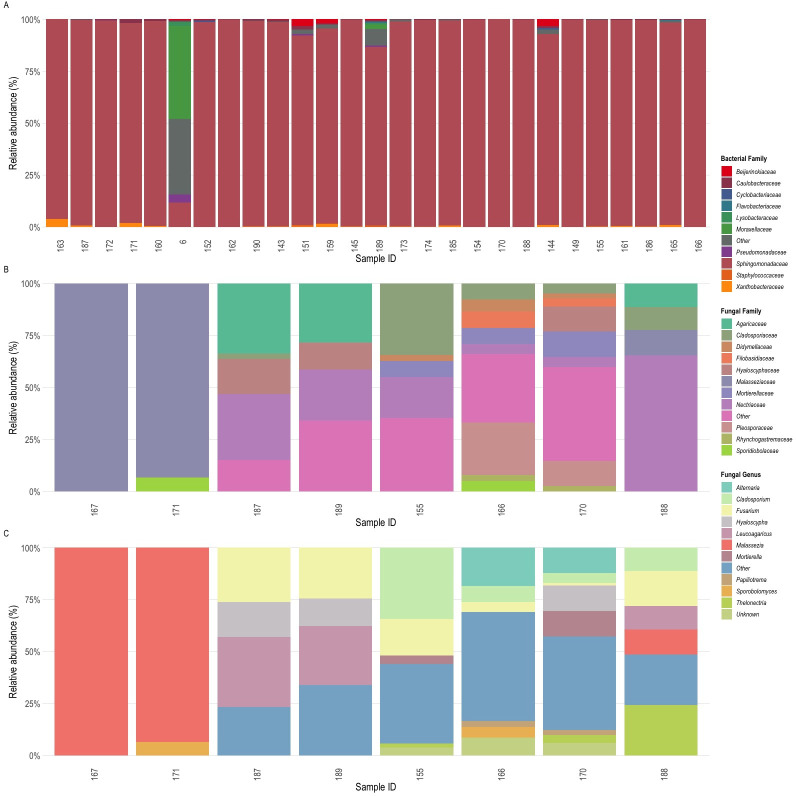
Bacterial and fungal relative abundances of the *Z. marina* seed microbiome. (**A**) Relative abundance of bacterial ASVs aggregated by family. Bacterial ASVs with less than 5% prevalence across samples were aggregated into the group “other”. Relative abundance of fungal ASVs aggregated by family (**B**) and by genus (**C**). Fungal ASVs with less than 15% prevalence across samples were aggregated into the group “other”. All relative abundances were calculated with the microbiome package (v1.20.0) (29). Sample IDs on the X-axis for all plots correspond to the IMR ID in Table 1.

For bacterial analysis, we generated 2,547,219 16S rRNA reads. Amplicon sequence variants (ASVs) were taxonomically assigned using SILVA (v138.1) (20, 21). ASVs were removed if assigned to chloroplast, mitochondrial, or plant sequences; unassigned at the domain level; identified as contamination; or had fewer than two reads per ASV. Samples with no matching ASVs or fewer than 1,000 reads were excluded from the data set. The final data set had 110 unique ASVs across 27 samples. Sphingomonadaceae was the most abundant family, representing 11.8% to 100% of the bacterial community, with ASVs primarily assigned to *Sphingomonas* (Fig. 1A). Within this genus, many isolates promote germination and growth in terrestrial plants (22, 23). Some *Sphingomonas* species alleviate salinity stress in terrestrial and wetland plants by altering osmotic metabolism pathways (24–26), which could benefit *Z. marina,* as germination requires low salinity to trigger water retention (27, 28).

**TABLE 1:** Metadata and sequencing information for all samples

IMR_ID	ENA accession (16S)	ENA accession (ITS2)	Seed_ID	Depth	Family within depth	Tissue type	16S rRNA amplicon	16S rRNA amplicon read input	16S rRNA amplicon filtered reads	16S rRNA amplicon denoised forward reads	16S rRNA amplicon denoised reverse reads	16S rRNA amplicon merged reads	16S rRNA amplicon nonchimeras	Sample kept for downstream 16S analysis	ITS2 amplicon	ITS2 amplicon input reads	ITS2 amplicon filtered reads	ITS2 amplicon denoised forward reads	ITS2 amplicon denoised reads	ITS2 amplicon merged reads	ITS2 amplicon nonchimeras	Sample kept for downstream ITS2 analysis
1	SAMEA120400796	–^*a*^	DCD-2-7	D	D_2	Seed	×^*b*^	3,536	3,126	3,101	3,103	2,583	2,583	–	–	–	–	–	–	–	–	–
3	SAMEA120400797	–	CHELEX-CON	NA	NA	Negative control	×	384	320	299	303	289	289	–	–	–	–	–	–	–	–	–
4	SAMEA120400798	–	DCS-6-8	S	S_6	Seed	×	26,852	24,030	23,945	23,978	20,623	20,623	–	–	–	–	–	–	–	–	–
5	SAMEA120400799	–	DCS-7-5	S	S_7	Seed	×	29,965	26,950	26,897	26,917	24,281	24,281	–	–	–	–	–	–	–	–	–
6	SAMEA120400800	–	DCD-8-6	D	D_8	Seed	×	17,603	14,249	14,000	14,105	13,097	13,051	×	–	–	–	–	–	–	–	–
7	SAMEA120400801	–	DCS-3-3	S	S_3	Seed	×	372	306	285	280	234	234	–	–	–	–	–	–	–	–	–
8	SAMEA120400802	–	DCD-2-6	D	D_2	Seed	×	29,769	26,447	26,411	26,430	23,918	23,917	–	–	–	–	–	–	–	–	–
10	SAMEA120400803	–	DCS-5-9	S	S_5	Seed	×	631	520	496	498	431	431	–	–	–	–	–	–	–	–	–
11	SAMEA120400804	–	DCD-2-3	D	D_2	Seed	×	28,705	25,818	25,798	25,808	23,347	23,346	–	–	–	–	–	–	–	–	–
12	SAMEA120400805	–	DCD-5-10	D	D_5	Seed	×	7,454	6,760	6,736	6,739	5,989	5,988	–	–	–	–	–	–	–	–	–
13	SAMEA120400806	–	DCM-2-3	M	M_2	Seed	×	20,458	18,469	18,440	18,450	15,776	15,776	–	–	–	–	–	–	–	–	–
14	SAMEA120400807	–	DCD-8-7	D	D_8	Seed	×	29,438	26,351	26,306	26,319	23,942	23,940	–	–	–	–	–	–	–	–	–
15	SAMEA120400808	–	DCS-3-4	S	S_3	Seed	×	13,996	12,813	12,803	12,806	11,552	11,552	–	–	–	–	–	–	–	–	–
16	SAMEA120400809	–	DCD-2-5	D	D_2	Seed	×	6,154	5,474	5,441	5,424	4,610	4,610	–	–	–	–	–	–	–	–	–
18	SAMEA120400810	–	DCD-4-6	D	D_4	Seed	×	55,296	50,110	50,061	50,076	45,433	45,421	–	–	–	–	–	–	–	–	–
20	SAMEA120400811	–	DCM-2-10	M	M_2	Seed	×	5,310	4,731	4,721	4,723	3,895	3,895	–	–	–	**–**	–	–	–	–	–
22	SAMEA120400812	–	DCD-8-8	D	D_8	Seed	×	15,974	14,316	14,289	14,292	12,808	12,808	–	–	–	–	–	–	–	–	–
23	SAMEA120400813	–	DCS-9-8	S	S_9	Seed	×	606	532	513	511	453	453	–	–	–	–	–	–	–	–	–
24	SAMEA120400814	–	DCS-6-6	S	S_6	Seed	×	16,040	14,212	14,154	14,169	12,059	12,059	–	–	–	–	–	–	–	–	–
25	SAMEA120400815	–	DCD-2-10	D	D_2	Seed	×	10,196	9,239	9,215	9,223	8,289	8,289	–	–	–	–	–	–	–	–	–
26	SAMEA120400816	–	DCD-6-4	D	D_6	Seed	×	132	64	53	58	53	53	–	–	–	–	–	–	–	–	–
27	SAMEA120400817	–	DCS-6-7	S	S_6	Seed	×	20,574	15,285	15,258	15,263	12,715	12,715	–	–	–	–	–	-	–	–	–
28	SAMEA120400818	–	DCS-6-2	S	S_6	Seed	×	20,977	18,939	18,867	18,889	16,430	16,430	–	–	–	–	–	–	–	–	–
29	SAMEA120400819	–	DCM-2-5	M	M_2	Seed	×	1,844	387	387	387	324	32	–	–	–	–	–	–	–	–	–
30	SAMEA120400820	–	DCD-8-10	D	D_8	Seed	×	47,812	42,498	42,434	42,451	38,789	38,716	–	–	–	–	–	–	–	–	–
31	SAMEA120400821	–	DCM-3-1	M	M_3	Seed	×	3,900	3,536	3,515	3,519	2,883	2,883	–	–	–	–	–	–	–	–	–
32	SAMEA120400822	–	DCD-2-2	D	D_2	Seed	×	1,584	1,355	1,322	1,323	1,076	1,076	–	–	–	–	–	–	–	–	–
33	SAMEA120400823	–	DCD-10-5	D	D_10	Seed	×	31,937	29,216	29,174	29,185	27,219	27,219	–	–	–	–	–	–	–	–	–
34	SAMEA120400824	–	DCD-10-10	D	D_10	Seed	×	15,299	13,858	13,820	13,840	12,112	12,112	–	–	–	–	–	–	–	–	–
36	SAMEA120400825	–	DCS-4-4	S	S_4	Seed	×	19,045	17,396	17,379	17,390	15,127	15,127	–	–	–	–	–	–	–	–	–
37	SAMEA120400826	–	DCS-7-9	S	S_7	Seed	×	33,487	29,163	29,122	29,122	24,995	24,994	–	–	–	–	–	–	–	–	–
38	SAMEA120400827	–	DCD-10-1	D	D_10	Seed	×	17,830	16,141	16,094	16,100	13,901	13,901	–	–	–	–	–	–	–	–	–
39	SAMEA120400828	–	DCS-3-7	S	S_3	Seed	×	394	334	313	320	283	283	–	–	–	–	–	–	–	–	–
40	SAMEA120400829	–	DCD-2-1	D	D_2	Seed	×	3,667	3,180	3,159	3,162	2,655	2,655	–	–	–	–	–	–	–	–	–
41	SAMEA120400830	–	DCS-10-2	S	S_10	Seed	×	21,783	19,724	19,699	19,701	17,118	17,118	–	–	–	–	–	–	–	–	–
42	SAMEA120400831	–	DCM-2-2	M	M_2	Seed	×	22,824	20,439	20,382	20,394	17,880	17,880	–	–	–	–	–	–	–	–	–
43	SAMEA120400832	–	DCS-4-5	S	S_4	Seed	×	7,944	7,124	7,105	7,108	6,115	6,115	–	–	–	–	–	–	–	–	–
44	SAMEA120400833	–	DCD-7-4	D	D_7	Seed	×	59,648	53,367	53,316	53,326	48,316	48,303	–	–	–	–	–	–	–	–	–
45	SAMEA120400834	–	DCS-9-9	S	S_9	Seed	×	170	133	119	122	104	104	–	–	–	–	–	–	–	–	–
46	SAMEA120400835	–	DCD-10-2	D	D_10	Seed	×	51,140	43,738	43,704	43,714	40,183	39,935	–	–	–	–	–	–	–	–	–
47	SAMEA120400836	–	DCS-3-8	S	S_3	Seed	×	461	388	362	374	312	307	–	–	–	–	–	–	–	–	–
48	SAMEA120400837	–	DCD-2-8	D	D_2	Seed	×	24,717	22,034	22,009	22,015	19,622	19,621	–	–	–	–	–	–	–	–	–
49	SAMEA120400838	–	DCS-10-3	S	S_10	Seed	×	10,527	9,337	9,286	9,290	8,096	8,096	–	–	–	–	–	–	–	–	–
50	SAMEA120400839	–	DCD-7-9	D	D_7	Seed	×	15,102	13,519	13,461	13,470	12,431	12,431	–	–	–	–	–	–	–	–	–
51	SAMEA120400840	–	DCD-8-4	D	D_8	Seed	×	43,000	38,887	38,828	38,856	35,485	35,483	–	–	–	–	–	–	–	–	–
52	SAMEA120400841	–	DCS-1-6	S	S_1	Seed	×	3,020	2,706	2,683	2,692	2,353	2,353	–	–	–	–	–	–	–	–	–
53	SAMEA120400842	–	DCS-5-1	S	S_5	Seed	×	4,073	3,621	3,595	3,607	3,167	3,167	–	–	–	–	–	–	–	–	–
54	SAMEA120400843	–	DCD-4-2	D	D_4	Seed	×	10,742	9,461	9,434	9,442	8,264	8,264	–	–	–	–	–	–	–	–	–
55	SAMEA120400844	–	DCD-6-7	D	D_6	Seed	×	1,026	903	876	880	742	742	–	–	–	–	–	–	–	–	–
56	SAMEA120400845	–	DCD-10-4	D	D_10	Seed	×	28,270	25,243	25,193	25,207	22,696	22,693	–	–	–	–	–	–	–	–	–
57	SAMEA120400846	–	DCS-5-4	S	S_5	Seed	×	1,009	861	842	849	724	724	–	–	–	–	–	–	–	–	–
58	SAMEA120400847	–	DCD-8-9	D	D_8	Seed	×	43,956	39,773	39,696	39,712	36,391	36,389	–	–	–	–	–	–	–	–	–
59	SAMEA120400848	–	DCS-4-6	S	S_4	Seed	×	13,071	11,838	11,810	11,821	10,008	10,008	–	–	–	–	–	–	–	–	–
60	SAMEA120400849	–	DCS-1-7	S	S_1	Seed	×	8,290	7,492	7,474	7,478	6,673	6,672	–	–	–	–	–	–	–	–	–
61	SAMEA120400850	–	DCS-9-2	S	S_9	Seed	×	351	295	274	273	217	217	–	–	–	–	–	–	–	–	–
62	SAMEA120400851	–	DCD-4-5	D	D_4	Seed	×	5,569	4,825	4,804	4,806	3,969	3,969	–	–	–	–	–	–	–	–	–
63	SAMEA120400852	–	DCD-6-8	D	D_6	Seed	×	542	490	476	475	445	445	–	–	–	–	–	–	–	–	–
64	SAMEA120400853	–	DCD-10-3	D	D_10	Seed	×	148,899	133,444	133,342	133,381	121,870	121,698	–	–	–	–	–	–	–	–	–
65	SAMEA120400854	–	DCD-4-3	D	D_4	Seed	×	21,642	19,134	19,099	19,115	17,309	17,303	–	–	–	–	–	–	–	–	–
66	SAMEA120400855	–	DCD-7-7	D	D_7	Seed	×	20,668	18,249	18,145	18,222	16,130	16,130	–	–	–	–	–	–	–	–	–
67	SAMEA120400856	–	DCS-10-1	S	S_10	Seed	×	41,196	37,012	36,967	36,981	32,876	32,816	–	–	–	–	–	–	–	–	–
68	SAMEA120400857	–	DCS-1-8	S	S_1	Seed	×	2,386	2,115	2,099	2,098	1,810	1,810	–	–	–	–	–	–	–	–	–
69	SAMEA120400858	–	DCD-7-10	D	D_7	Seed	×	29,641	26,822	26,789	26,803	24,491	24,491	–	–	–	–	–	–	–	–	–
70	SAMEA120400859	–	DCS-5-5	S	S_5	Seed	×	553	459	442	440	372	372	–	–	–	–	–	–	–	–	–
71	SAMEA120400860	–	DCD-6-9	D	D_6	Seed	×	1,391	1,236	1,219	1,221	1,100	1,100	–	–	–	–	–	–	–	–	–
72	SAMEA120400861	–	DCD-10-8	D	D_10	Seed	×	36,678	32,783	32,736	32,732	29,672	29,638	–	–	–	–	–	–	–	–	–
73	SAMEA120400862	–	DCD-4-4	D	D_4	Seed	×	21,740	19,343	19,320	19,327	16,799	16,799	–	–	–	–	–	–	–	–	–
74	SAMEA120400863	–	DCS-4-10	S	S_4	Seed	×	3,699	3,109	3,095	3,096	2,675	2,675	–	–	–	–	–	–	–	–	–
75	SAMEA120400864	–	DCS-9-10	S	S_9	Seed	×	739	657	644	642	581	581	–	–	–	–	–	–	–	–	–
76	SAMEA120400865	–	DCS-1-9	S	S_1	Seed	×	3,696	3,323	3,305	3,305	2,812	2,812	–	–	–	–	–	–	–	–	–
77	SAMEA120400866	–	DCS-6-3	S	S_6	Seed	×	12,859	11,540	11,501	11,515	9,808	9,808	–	–	–	–	–	–	–	–	–
78	SAMEA120400867	–	DCD-4-10	D	D_4	Seed	×	18,966	16,716	16,684	16,695	13,497	13,497	–	–	–	–	–	–	–	–	–
79	SAMEA120400868	–	DCD-6-10	D	D_6	Seed	×	268	226	209	210	206	206	–	–	–	–	–	–	–	–	–
80	SAMEA120400869	–	DCD-10-9	D	D_10	Seed	×	7,989	6,235	6,087	6,131	5,383	5,383	–	–	–	–	–	–	–	–	–
81	SAMEA120400870	–	DCD-5-1	D	D_5	Seed	×	6,109	5,454	5,430	5,430	4,808	4,808	–	–	–	–	–	–	–	–	–
82	SAMEA120400871	–	DCS-6-4	S	S_6	Seed	×	7,744	6,933	6,908	6,914	5,826	5,826	–	–	–	–	–	–	–	–	–
83	SAMEA120400872	–	DCS-5-8	S	S_5	Seed	×	550	474	445	446	396	396	–	–	–	–	–	–	–	–	–
84	SAMEA120400873	–	DCS-7-3	S	S_7	Seed	×	19,521	17,456	17,429	17,432	14,908	14,908	–	–	–	–	–	–	–	–	–
85	SAMEA120400874	–	DCM-2-4	M	M_2	Seed	×	2,590	2,329	2,309	2,317	2,052	2,052	–	–	–	–	–	–	–	–	–
86	SAMEA120400875	–	DCS-3-1	S	S_3	Seed	×	5,017	4,401	4,382	4,389	3,832	3,832	–	–	–	–	–	–	–	–	–
87	SAMEA120400876	–	DCD-4-1	D	D_4	Seed	×	8,375	7,456	7,437	7,421	6,351	6,351	–	–	–	–	–	–	–	–	–
88	SAMEA120400877	–	DCD-6-1	D	D_6	Seed	×	801	686	671	673	603	603	–	–	–	–	–	–	–	–	–
89	SAMEA120400878	–	DCD-5-2	D	D_5	Seed	×	2,955	2,676	2,659	2,653	2,355	2,355	–	–	–	–	–	–	–	–	–
91	SAMEA120400879	–	DCD-5-3	D	D_5	Seed	×	6,390	5,805	5,783	5,778	5,072	5,072	–	–	–	–	–	–	–	–	–
92	SAMEA120400880	–	DCS-7-4	S	S_7	Seed	×	15,546	14,003	13,962	13,970	12,413	12,413	–	–	–	–	–	–	–	–	–
93	SAMEA120400881	–	DCS-7-8	S	S_7	Seed	×	12,032	10,918	10,897	10,906	9481	9481	–	–	–	–	–	–	–	–	–
94	SAMEA120400882	–	DCS-3-2	S	S_3	Seed	×	17,274	15,396	15,338	15,354	13,869	13,869	–	–	–	–	–	–	–	–	–
95	SAMEA120400883	–	DCD-2-9	D	D_2	Seed	×	9,819	8,927	8,911	8,917	7,662	7,662	–	–	–	–	–	–	–	–	–
96	SAMEA120400884	–	DCD-7-8	D	D_7	Seed	×	2,874	2,580	2,549	2,550	2,351	2,351	–	–	–	–	–	–	–	–	–
98	SAMEA120400885	–	DCS-10-6	S	S_10	Seed	×	15,563	13,989	13,967	13,966	11,516	11,516	–	–	–	–	–	–	–	–	–
99	SAMEA120400886	–	DCD-7-3	D	D_7	Seed	×	41,894	37,853	37,817	37,830	35,621	35,611	–	–	–	–	–	–	–	–	–
100	SAMEA120400887	–	DCD-4-8	D	D_4	Seed	×	2,375	2,091	2,073	2,074	1,886	1,886	–	–	–	–	–	–	–	–	–
101	SAMEA120400888	–	DCD-6-3	D	D_6	Seed	×	1,433	1,245	1,225	1,230	1,055	1,055	–	–	–	–	–	–	–	–	–
102	SAMEA120400889	–	DCS-9-1	S	S_9	Seed	×	699	586	558	556	481	481	–	–	–	–	–	–	–	–	–
103	SAMEA120400890	–	DCM-5-9	M	M_5	Seed	×	3,638	3,142	3,015	3,096	2,719	2,719	–	–	–	–	–	–	–	–	–
104	SAMEA120400891	–	DCD-7-5	D	D_7	Seed	×	2,154	1,914	1,907	1,906	1,652	1,652	–	–	–	–	–	–	–	–	–
107	SAMEA120400892	–	DCS-9-3	S	S_9	Seed	×	376	316	302	303	262	262	–	–	–	–	–	–	–	–	–
108	SAMEA120400893	–	DCS-4-9	S	S_4	Seed	×	28,471	25,784	25,751	25,762	22,430	22,427	–	–	–	–	–	–	–	–	–
109	SAMEA120400894	–	DCD-4-9	D	D_4	Seed	×	39,973	35,899	35,867	35,880	33,256	33,253	–	–	–	–	–	–	–	–	–
110	SAMEA120400895	–	DCS-4-3	S	S_4	Seed	×	5,259	4,475	4,449	4,464	3,872	3,872	–	–	–	–	–	–	–	–	–
111	SAMEA120400896	–	DCM-5-10	M	M_5	Seed	×	2,322	1,618	1,547	1,543	1,310	1,310	–	–	–	–	–	–	–	–	–
112	SAMEA120400897	–	DCM-3-2	M	M_3	Seed	×	31,051	28,003	27,967	27,986	24,930	24,930	–	–	–	–	–	–	–	–	–
113	SAMEA120400898	–	DCS-1-1	S	S_1	Seed	×	204	151	120	125	96	96	–	–	–	–	–	–	–	–	–
114	SAMEA120400899	–	DCS-10-8	S	S_10	Seed	×	21,315	19,023	18,978	18,975	15,317	15,317	–	–	–	–	–	–	–	–	–
115	SAMEA120400900	–	DCD-8-1	D	D_8	Seed	×	36,288	32,147	32,076	32,100	29,265	29,265	–	–	–	–	–	–	–	–	–
116	SAMEA120400901	–	DCD-6-2	D	D_6	Seed	×	349	293	283	283	240	240	–	–	–	–	–	–	–	–	–
117	SAMEA120400902	–	DCS-10-9	S	S_10	Seed	×	39,544	34,600	34,553	34,570	30,038	30,022	–	–	–	–	–	–	–	–	–
118	SAMEA120400903	–	DCD-10-7	D	D_10	Seed	×	48,364	41,965	41,929	41,881	38,449	38,294	–	–	–	–	–	–	–	–	–
119	SAMEA120400904	–	DCM-6-1	M	M_6	Seed	×	497	398	386	383	361	361	–	–	–	–	–	–	–	–	–
120	SAMEA120400905	–	DCS-5-3	S	S_5	Seed	×	115	91	76	86	76	76	–	–	–	–	–	–	–	–	–
121	SAMEA120400906	–	DCS-1-2	S	S_1	Seed	×	579	494	475	478	421	421	–	–	–	–	–	–	–	–	–
122	SAMEA120400907	–	DCM-2-7	M	M_2	Seed	×	12,806	11,683	11,660	11,665	10,517	10,517	–	–	–	–	–	–	–	–	–
123	SAMEA120400908	–	DCS-6-5	S	S_6	Seed	×	83	55	47	48	47	47	–	–	–	–	–	–	–	–	–
124	SAMEA120400909	–	DCS-4-1	S	S_4	Seed	×	47,751	43,496	43,475	43,483	39,981	39,945	–	–	–	–	–	–	–	–	–
125	SAMEA120400910	–	DCS-7-6	S	S_7	Seed	×	31,064	27,597	27,558	27,577	24,521	24,513	–	–	–	–	–	–	–	–	–
126	SAMEA120400911	–	DCD-8-2	D	D_8	Seed	×	523	479	471	471	437	437	–	–	–	–	–	–	–	–	–
127	SAMEA120400912	–	DCM-6-2	M	M_6	Seed	×	681	557	535	535	465	465	–	–	–	–	–	–	–	–	–
128	SAMEA120400913	–	DCD-10-6	D	D_10	Seed	×	26,536	23,889	23,808	23,838	21,905	21,858	–	–	–	–	–	–	–	–	–
129	SAMEA120400914	–	DCS-1-3	S	S_1	Seed	×	1,345	1,201	1,174	1,170	1,021	1,021	–	–	–	–	–	–	–	–	–
130	SAMEA120400915	–	DCS-10-10	S	S_10	Seed	×	10,181	9,125	9,094	9,086	7,849	7,849	–	–	–	–	–	–	–	–	–
131	SAMEA120400916	–	DCS-9-4	S	S_9	Seed	×	100	65	57	60	57	57	–	–	–	–	–	–	–	–	–
132	SAMEA120400917	–	DCD-5-5	D	D_5	Seed	×	8,117	7,203	7,189	7,189	6,395	6,395	–	–	–	–	–	–	–	–	–
133	SAMEA120400918	–	DCS-6-9	S	S_6	Seed	×	234	175	156	158	145	145	–	–	–	–	–	–	–	–	–
134	SAMEA120400919	–	DCS-3-6	S	S_3	Seed	×	365	327	316	319	302	302	–	–	–	–	–	–	–	–	–
135	SAMEA120400920	–	DCM-6-3	M	M_6	Seed	×	446	351	330	340	330	330	–	–	–	–	–	–	–	–	–
136	SAMEA120400921	–	DCM-2-6	M	M_2	Seed	×	9,336	8,432	8,415	8,422	7,366	7,366	–	–	–	–	–	–	–	–	–
137	SAMEA120400922	–	DCS-1-4	S	S_1	Seed	×	760	645	627	630	515	515	–	–	–	–	–	–	–	–	–
139	SAMEA120400923	–	DCD-2-4	D	D_2	Seed	×	3,167	2,822	2,812	2,814	2,442	2,442	–	–	–	–	–	–	–	–	–
140	SAMEA120400924	–	DCD-7-6	D	D_7	Seed	×	27,416	24,777	24,745	24,755	22,365	22,364	–	–	–	–	–	–	–	–	–
141	SAMEA120400925	–	DCS-3-10	S	S_3	Seed	×	57,731	51,221	51,177	51,198	44,958	44,890	–	–	–	–	–	–	–	–	–
142	SAMEA120400926	–	DCS-7-1	S	S_7	Seed	×	20,177	18,293	18,245	18,288	16,003	16,002	-–	–	–	–	–	–	–	–	–
143	SAMEA120400927	SAMEA120400970	DCM-6-4	M	M_6	Seed	×	37,549	33,456	33,410	33,426	29,580	29,579	×	×	6,320	5,333	5,326	5,323	5,277	5,277	–
144	SAMEA120400928	SAMEA120400971	DCS-5-6	S	S_5	Seed	×	1,789	1,556	1,542	1,543	1,537	1,537	×	×	239	142	131	131	131	131	–
145	SAMEA120400929	SAMEA120400972	DCS-1-5	S	S_1	Seed	×	11,012	9,750	9,732	9,737	9,731	9,731	×	×	561	439	420	431	420	420	–
147	SAMEA120400930	SAMEA120400973	DCD-5-8	D	D_5	Seed	×	9,413	8,540	8,524	8,532	7,669	7,669	–	×	1,240	1,012	1,007	1,007	1,007	1,007	–
148	SAMEA120400931	SAMEA120400974	DCD-5-6	D	D_5	Seed	×	13,702	12,435	12,412	12,419	11,048	11,048	–	×	4,407	3,787	3,783	3,783	3,782	3,782	–
149	SAMEA120400932	SAMEA120400975	DCS-5-2	S	S_5	Seed	×	24,206	21,036	21,012	20,999	20,200	20,200	×	×	6,056	5,100	5,089	5,087	5,087	5,087	–
150	SAMEA120400933	SAMEA120400976	DCS-9-5	S	S_9	Seed	×	772	676	655	655	638	638	–	×	206	133	112	113	91	91	–
151	SAMEA120400934	SAMEA120400977	DCM-6-5	M	M_6	Seed	×	1,638	1,401	1,383	1,389	1,383	1,383	×	×	120	31	23	23	23	23	–
152	SAMEA120400935	SAMEA120400978	DCM-2-9	M	M_2	Seed	×	25,077	22,679	22,663	22,669	20,259	20,258	×	×	11,552	9,796	9,761	9,778	9,753	9,753	–
153	SAMEA120400936	SAMEA120400979	DCD-8-5	D	D_8	Seed	×	20,807	18,854	18,807	18,828	16,759	16,759	–	×	20,599	17,511	17,485	17,503	17,484	17,484	–
154	SAMEA120400937	SAMEA120400980	DCS-3-9	S	S_3	Seed	×	18,938	16,987	16,967	16,970	15,470	15,470	×	×	10,067	8,591	8,582	8,526	8,520	8,520	×
155	SAMEA120400938	SAMEA120400981	DCS-5-7	S	S_5	Seed	×	26,849	23,719	23,539	23,594	23,441	23,441	×	×	1,472	1,068	1,063	1,063	1,063	1,063	–
156	SAMEA120400939	SAMEA120400982	DCD-7-2	D	D_7	Seed	×	50,849	45,948	45,915	45,929	40,737	40,737	–	×	22,437	19,317	19,313	19,312	19,312	19,312	–
158	SAMEA120400940	SAMEA120400983	DCS-3-5	S	S_3	Seed	×	4,081	3,708	3,703	3,703	3,238	3,238	–	×	3,144	2,630	2,624	2,624	2,594	2,594	–
159	SAMEA120400941	SAMEA120400984	DCM-6-6	M	M_6	Seed	×	1,551	1,337	1,331	1,330	1,328	1,328	×	×	99	44	40	40	40	40	–
160	SAMEA120400942	SAMEA120400985	DCD-7-1	D	D_7	Seed	×	21,756	19,636	19,619	19,627	18,538	18,538	×	×	7,788	6,589	6,534	6,583	6,533	6,533	–
161	SAMEA120400943	SAMEA120400986	DCS-6-10	S	S_6	Seed	×	9,572	8,475	8,461	8,464	8,128	8,128	×	×	5,613	4,706	4,696	4,699	4,696	4,696	–
162	SAMEA120400944	SAMEA120400987	DCM-2-8	M	M_2	Seed	×	8,533	7,608	7,597	7,601	7,059	7,059	×	×	587	465	461	461	461	461	–
163	SAMEA120400945	SAMEA120400988	DCD-4-11	D	D_4	Seed	×	27,328	24,488	24,460	24,472	22,221	22,221	×	×	8,022	6,675	6,671	6,669	6,667	6,667	–
165	SAMEA120400946	SAMEA120400989	DCS-7-7	S	S_7	Seed	×	15,853	13,737	13,625	13,668	13,323	13,309	×	×	1,589	1,270	1,267	1.267	1,267	1,267	×
166	SAMEA120400947	SAMEA120400990	DCS-7-10	S	S_7	Seed	×	33,121	29,445	29,430	29,440	29,237	29,237	×	×	7,570	6,370	6,352	6,352	6,290	6,290	×
167	SAMEA120400948	SAMEA120400991	DCM-6-7	M	M_6	Seed	×	375	272	250	262	237	237	–	×	1,697	1,290	1,265	1,289	1,256	1,256	–
168	SAMEA120400949	SAMEA120400992	DCS-9-6	S	S_9	Seed	×	599	528	504	516	492	492	–	×	283	236	233	233	233	233	–
169	SAMEA120400950	SAMEA120400993	DCS-9-7	S	S_9	Seed	×	241	206	196	198	175	175	–	×	88	57	54	54	54	54	×
170	SAMEA120400951	SAMEA120400994	DCS-4-7	S	S_4	Seed	×	10,144	9,114	9,103	9,101	8,479	8,479	×	×	2,243	1,879	1,836	1,843	1,815	1,815	×
171	SAMEA120400952	SAMEA120400995	DCD-6-6	D	D_6	Seed	×	1,370	1,210	1,196	1,206	1,196	1,196	×	×	1,474	1,134	1,133	1,132	1,112	1,112	–
172	SAMEA120400953	SAMEA120400996	DCD-5-9	D	D_5	Seed	×	5,424	4,888	4,869	4,876	4,452	4,452	×	×	782	610	604	604	604	604	–
173	SAMEA120400954	SAMEA120400997	DCS-10-7	S	S_10	Seed	×	17,654	15,348	15,300	15,322	12,834	12,834	×	×	4,832	4,125	4,117	4,117	4,073	4,073	–
174	SAMEA120400955	SAMEA120400998	DCS-10-4	S	S_10	Seed	×	15,898	14,231	14,216	14,224	14,194	14,194	×	×	1,456	1,205	1,192	1,193	1,192	1,192	–
175	SAMEA120400956	SAMEA120400999	DCM-6-8	M	M_6	Seed	×	388	335	328	327	322	322	–	×	305	194	190	190	177	177	–
176	SAMEA120400957	SAMEA120401000	DCD-8-3	D	D_8	Seed	×	19,130	17,291	17,241	17,258	15,595	15,595	–	×	4,908	4,090	4,080	4,082	4,079	4,079	–
177	SAMEA120400958	SAMEA120401001	DCS-7-2	S	S_7	Seed	×	10,629	9,580	9,532	9,505	8,094	8,094	–	×	4,727	3,967	3,950	3,954	3,950	3,950	–
178	SAMEA120400959	SAMEA120401002	DCS-4-8	S	S_4	Seed	×	2,406	2,158	2,138	2,138	1,834	1,833	–	×	482	378	357	364	355	355	–
179	SAMEA120400960	SAMEA120401003	DCS-5-10	S	S_5	Seed	×	4,936	4,024	4,000	4,010	3,439	3,439	–	×	1,467	1,209	1,202	1,204	1,202	1,202	–
180	SAMEA120400961	SAMEA120401004	DCD-6-5	D	D_6	Seed	×	339	300	293	295	281	281	–	×	121	72	70	70	70	70	–
182	SAMEA120400962	SAMEA120401005	DCM-5-7	M	M_5	Seed	×	6,009	5,451	5,441	5,437	4,723	4,723	–	×	648	532	526	526	526	526	–
183	SAMEA120400963	SAMEA120401006	DCM-3-3	M	M_3	Seed	×	22,931	20,420	20,403	20,407	17,660	17,660	–	×	10,156	8,625	8,617	8,617	8,516	8,516	–
185	SAMEA120400964	SAMEA120401007	DCS-10-5	S	S_10	Seed	×	13,914	12,467	12,420	12,397	11,162	11,162	×	×	2,387	1,955	1,939	1,946	1,921	1,921	–
186	SAMEA120400965	SAMEA120401008	DCS-6-1	S	S_6	Seed	×	15,019	13,108	13,095	13,099	12,755	12,755	×	×	3,425	2,895	2,887	2,887	2,887	2,887	×
187	SAMEA120400966	SAMEA120401009	DCD-4-7	D	D_4	Seed	×	18,521	16,439	16,422	16,429	16,220	16,220	×	×	3,003	2,452	2,446	2,443	2,443	2,443	×
188	SAMEA120400967	SAMEA120401010	DCS-4-2	S	S_4	Seed	×	13,421	11,599	11,580	11,582	11,489	11,489	×	×	4,659	3,884	3,879	3,879	3,827	3,827	×
189	SAMEA120400968	SAMEA120401011	DCS-1-10	S	S_1	Seed	×	5,221	4,599	4,523	4,564	4,407	4,407	×	×	9,515	8,072	8,060	8,059	8,012	8,012	–
190	SAMEA120400969	SAMEA120401012	DCM-5-8	M	M_5	Seed	×	11,909	10,592	10,529	10,548	10,035	10,035	×	×	6,420	5,430	5,414	5,416	5,411	5,411	–

^*a*^ –, not applicable to the sample.

^*b*^ x indicates the sample was processed for the corresponding amplicon sequencing method (16S or ITS2).

For fungi, we generated 184,766 ITS2 reads. ASVs were taxonomically assigned using the UNITE “all eukaryotes” general Fasta data set with *Z. marina* 5.8S ITS2 added (30). ASVs not assigned to fungi or with fewer than two reads per ASV were removed. Samples without matching fungal ASVs or with fewer than 100 reads per sample were excluded, yielding 60 unique ASVs in 8 samples. Nectriaceae was the most abundant family, representing up to 65% of the fungal community, with ASVs primarily assigned to *Fusarium* (up to 26%; Fig. 1B and C). While *Fusarium* species are typically seed pathogens in terrestrial systems (31), they are predicted to be non-pathogenic and potentially beneficial for seagrasses (32, 33), highlighting contrasting ecosystem roles. Two samples were dominated by Malasseziaceae, with ASVs assigned to *Malassezia*. Given that *Malassezia* is widespread in marine environments (34), its dynamics with *Z. marina* seed development and mycobiome interactions warrant further investigation.

## Data Availability

Amplicon sequencing data for 16S rRNA gene and ITS have been deposited in the European Nucleotide Archive (ENA) under project accession number PRJEB100937.
